# Lung–infiltrating T helper 17 cells as the major source of interleukin-17A production during pulmonary *Cryptococcus neoformans* infection

**DOI:** 10.1186/s12865-018-0269-5

**Published:** 2018-11-08

**Authors:** Elaheh Movahed, Yi Ying Cheok, Grace Min Yi Tan, Chalystha Yie Qin Lee, Heng Choon Cheong, Rukumani Devi Velayuthan, Sun Tee Tay, Pei Pei Chong, Won Fen Wong, Chung Yeng Looi

**Affiliations:** 1 Department of Medical Microbiology, Faculty of Medicine, University of Malaya, Kuala Lumpur, Malaysia; 2 School of Bioscience, Taylor’s University, Subang Jaya, Selangor Malaysia

**Keywords:** CD4^+^ T cells, T_H_17 cells, Macrophages, IL-17A, *Cryptococcus neoformans*

## Abstract

**Background:**

IL-17A has emerged as a key player in the pathologies of inflammation, autoimmune disease, and immunity to microbes since its discovery two decades ago. In this study, we aim to elucidate the activity of IL-17A in the protection against *Cryptococcus neoformans*, an opportunistic fungus that causes fatal meningoencephalitis among AIDS patients. For this purpose, we examined if *C. neoformans* infection triggers IL-17A secretion in vivo using wildtype C57BL/6 mice. In addition, an enhanced green fluorescence protein (EGFP) reporter and a knockout (KO) mouse models were used to track the source of IL-17A secretion and explore the protective function of IL-17A, respectively.

**Results:**

Our findings showed that in vivo model of *C. neoformans* infection demonstrated induction of abundant IL-17A secretion. By examining the lung bronchoalveolar lavage fluid (BALF), mediastinal lymph node (mLN) and spleen of the IL-17A–EGFP reporter mice, we showed that intranasal inoculation with *C. neoformans* promoted leukocytes lung infiltration. A large proportion (~ 50%) of the infiltrated CD4^+^ helper T cell population secreted EGFP, indicating vigorous T_H_17 activity in the *C. neoformans*–infected lung. The infection study in IL-17A–KO mice, on the other hand, revealed that absence of IL-17A marginally boosted fungal burden in the lung and accelerated the mouse death.

**Conclusion:**

Therefore, our data suggest that IL-17A is released predominantly from T_H_17 cells in vivo, which plays a supporting role in the protective immunity against *C. neoformans* infection.

**Electronic supplementary material:**

The online version of this article (10.1186/s12865-018-0269-5) contains supplementary material, which is available to authorized users.

## Background

The opportunistic pathogenic basidiomycete *Cryptococcus neoformans* is an encapsulated yeast commonly found in bird excrement worldwide [1]. The infection is often asymptomatic in healthy individuals but causes severe pulmonary cryptococcosis and life-threatening meningoencephalitis in immunocompromised patients. *C. neoformans* has gained attention in recent years as it is a major cause of death among patients who have advanced acquired immunodeficiency syndrome (AIDS) [2]. Hence, it is important to study the host interaction with this pathogen as 30–60% of the patients who have cryptococcal meningitis succumb to cryptococcosis infection within 1 year despite antifungal therapy [3].

The essential role of T cells in the host immune response to *C. neoformans* has been well-studied using T cell depletion mouse model [4–6]. In recent years, T_H_17, has been implicated in the immune response to fungus such as *Candida albicans* [7, 8]. A different study for *Aspergillus fumigatus* claimed that IL-17A promotes the fungal infection [9]; as such, the nature and role of T_H_17 cell subset require further investigations. T_H_17 cell is characterized by its hallmark RORγt transcription factor and IL-17A secretion. Its differentiation from naïve CD4^+^ T cells is induced in the presence of IL-6 and TGF-β during inflammatory response. IL-23 is another important inducer for IL-17A as the IL-17A production was strongly impaired in the IL-23p19 deficient mice [10]. *C. gattii*, a highly virulent cryptococcal species is able to attenuate both T_H_1 and T_H_17 by suppressing *IL-12* and *IL-23* genes transcription [11].

T_H_17 is not the sole source of IL-17A as it can also be released by other cells such as macrophages, NK cells, and neutrophils [12]. In the mice with helper T cell impairment, *C. neoformans* infection caused a compensatory neutrophil response that required IL-17A [13], whereas in neutrophil-depleted mice, *C. neoformans* infection results in increase IL-17A and IL-17A^+^ γδ^+^ T cells [13]. IL-17A elicits inflammatory response by recruiting neutrophils, but does not contribute to classical macrophage activation as seen in pulmonary cryptococcosis induction in the mouse model [14]. IL-17A enhances host defense against lung infection with moderately virulent *C. neoformans* through leukocyte recruitment and activation besides inducing release of IFN-γ [15]. T_H_17 cells release a panel of other cytokines in addition to IL-17A such as IL-17F, IL-22, and IL-23. *C. neoformans* produces prostaglandin E2 (PGE2) which inhibits Interferon regulatory factor 4 (IRF4) and IL-17A but not IL-22 [15]. A full picture of regulatory mechanism as to how this subset of T cell interacts and eliminates the fungal infection requires further investigation.

In this study, we examined the association of IL-17A with *C. neoformans* infection by using in vivo infection model. The main focus of our study lies on identifying the source of IL-17A secretion and determining its protective role in *C. neoformans*–infected mice. Using enhanced green fluorescence protein (EGFP) reporter mouse model, we showed that lung infiltrating T_H_17 cells are likely the predominant source of IL-17A. Data from a knockout (KO) mouse model supports a protective function of the IL-17A against *C. neoformans* infection.

## Results

### *C. neoformans* infection induces IL-17A production in in vivo model

**Fig. 1:**
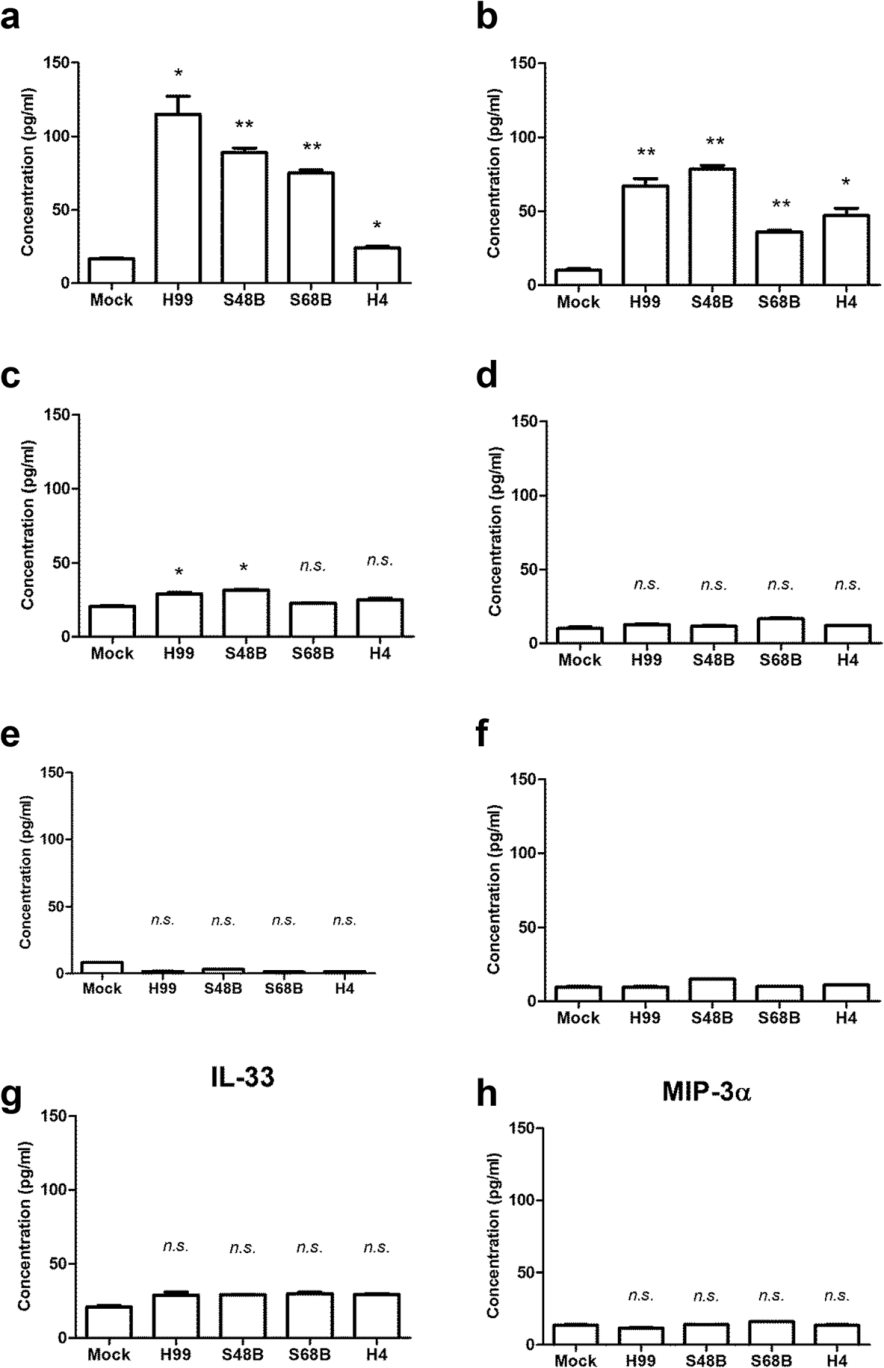
Elevated serum IL-17A, IL-23 and IL-17F levels in the *C. neoformans*–infected mice. C57BL/6 mice (*n* = 2 per group) were intranasally inoculated with 1 × 10^5^ cells of four different strains of *C. neoformans* (H99, S48B, S68B and H4), serum were collected after 14 days for Bio-plex cytokine array. Mock denotes control mice intranasally administrated with equal volume of PBS. Different cytokines in the T_H_17 panel, (**a**) IL-17A, (**b**) IL-17F, (**c**) IL-21, (**d**) IL-22, (**e**) IL-23, (**f**) IL-31, (**g**) IL-33 and (**h**) MIP-3α were measured. Data are representative of two independent experiments. *P < 0.05, **P < 0.01, *n.s.*: not significant or *P* ≥ 0.05, by Student’s *t*-test

To investigate if *C. neoformans* infection–mediated IL-17A secretion occurs in vivo, C57BL/6 mice were intranasally inoculated with four different strains of *C. neoformans* (H99, S48B, S68B and H4) at 2 × 10^5^ cells for 14 days. These *C. neoformans* strains exhibit distinct virulence properties as shown by different fungal burdens in a pulmonary infection mouse model. Such differences are attributed to variations in their transcriptome profile, ability in polysaccharide capsule formation as well as laccase activity [16]. Serum was collected for Bio-plex Pro Mouse Th17 assay which included the following cytokines: IL-17A, IL-17F, IL-21, IL-22, IL-23, IL-31, IL-33 and MIP-3α (Fig. 1). An elevated serum level of IL-17A was detected in the *C. neoformans*–infected mice (Fig. 1a). We noted that the serum IL-17A level was correlated with the degree of virulence of different *C. neoformans* strains [16], whereby the highest amount of serum IL-17A was observed in the group of mice infected with the most virulent *C. neoformans* H99 strain (115 ± 12 pg/ml). This was followed by moderate serum IL-17A level observed in the mice infected with less virulent environmental strains, S48S (89 ± 3 pg/ml) and S68B (75 ± 2 pg/ml), and lowest level of serum IL-17A was noted in the mice infected with non-virulent strain H4 (24 ± 1 pg/ml) strains, compared to control uninfected mice (< 20 pg/ml). The level of serum IL-23 was also elevated in all *C. neoformans*–infected mice, i.e. H99 (67 ± 5 pg/ml), S48S (78 ± 3 pg/ml), S68B (36 ± 1 pg/ml) and H4 (47 ± 5 pg/ml) compared to < 20 pg/ml in the mock control (Fig. 1b). This suggests IL-23–IL-17A axis pathway plays a major role in the host immunity against *C. neoformans* infection. On the other hand, the serum IL-17F levels were only scarcely increased in mice infected with *C. neoformans* H99 and S48B strains (Fig. 1c). Whereas no noteworthy induction was observed for other cytokines (IL-21, IL-22, IL-31 and IL-33, and MIP-3α) examined (Fig. 1d, e, f, g, h).

### Intranasal *C. neoformans* inoculation causes leukocytes lung infiltration

**Fig. 2:**
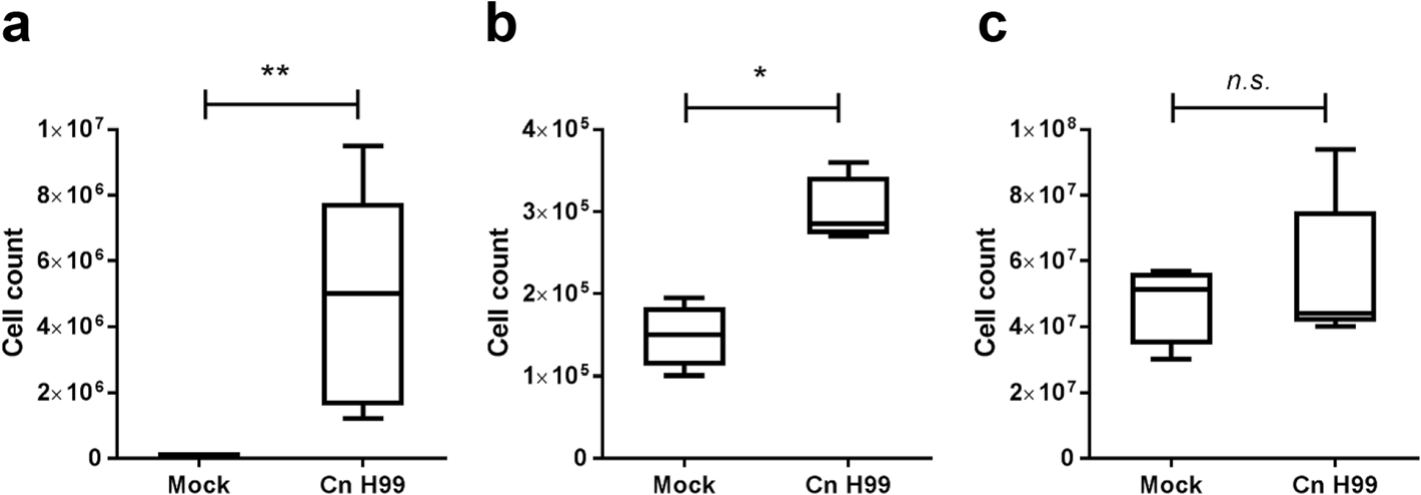
Increased number of leukocytes in BALF and mLN of the *C. neoformans*–infected mice. IL-17A–EGFP reporter mice (*n* = 4 per group) were uninfected (mock) or intranasally inoculated with 1 × 10^5^ cells with *C. neoformans* H99 strain (Cn H99), BALF, mLN and spleen were collected after 14 days for analysis. Total number of cells in (**a**) BALF, (**b**) mLN and (**c**) spleen were determined by manual cell count using a haemocytometer. *P < 0.05, **P < 0.01, *n.s.*: not significant or *P* ≥ 0.05, by Mann-Whitney U test

To examine the importance of IL-17A in providing immunity to *C. neoformans* infection, we utilized a mouse model harboring IRES-EGFP-SV40-polyA signal sequence cassette after the stop codon of *Il-17A* gene in which the EGFP is co-expressed in the IL-17A–producing cells. Mice were intranasally administrated with 20 μl of control PBS or *C. neoformans* (2 × 10^5^ cells) in suspension, and splenic, mediastinal lymph nodes (mLN), bronchoalveolar lavage fluid (BALF) cells were inspected after 4 weeks (Fig. 2). Total numbers of cells were significantly increased in the BALF (4.8 × 10^6^ ± 1.0 × 10^6^ versus 4.6 × 10^4^ ± 1.0 × 10^4^ cells) and mLN (3.0 × 10^5^ ± 2.0 × 10^4^ versus 1.5 × 10^5^ ± 1.9 × 10^4^ cells) of the *C. neoformans* H99–infected mice versus the control group (Fig. 2a, b). No significant increased numbers of cells were observed in the spleen after intranasal *C. neoformans* H99 infection (5.6 × 10^7^ ± 7.1 × 10^6^ versus 4.7 × 10^7^ ± 6.1 × 10^6^) (Fig. 2c).

**Fig. 3:**
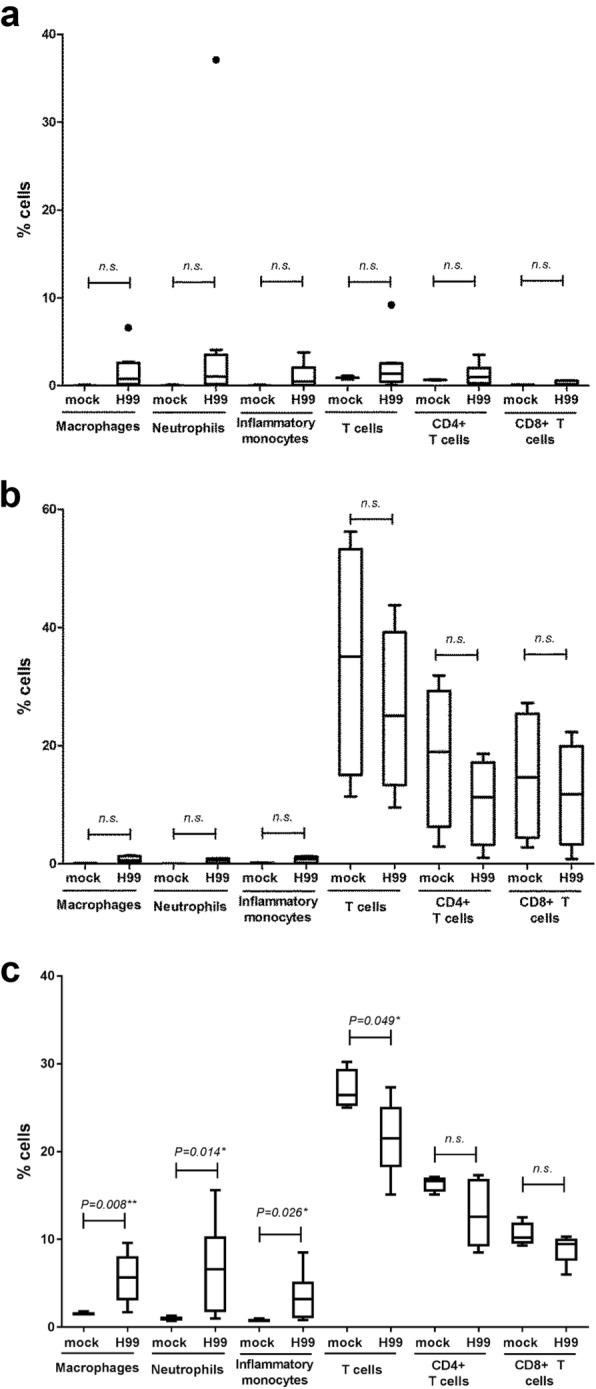
Percentages of leukocytes in spleen of the *C. neoformans*–infected mice. Box and whiskers plot show the percentages of different leukocytes in the (**a**) BALF, (**b**) mLN, and (**c**) spleen. IL-17A–EGFP reporter mice (*n* = 4 per group) were uninfected (mock) or intranasally inoculated with 1 × 10^5^ cells with *C. neoformans* H99 strain (Cn H99). Number of GFP^+^ cells among the macrophages (F4/80^+^ CD11b^+^ Gr1^+^)–, neutrophils (F4/80^−^ CD11b^+^ Gr-1^high^)–, and inflammatory monocytes (F4/80^−^ CD11b^+^ Gr1^medium^)–, T cells (TCRβ^+^)–, helper T cells (TCRβ^+^ CD4^+^)–, cytotoxic T cells (TCRβ^+^ CD8^+^)–gated cell populations. *P < 0.05, **P < 0.01, *n.s.*: not significant or *P* ≥ 0.05, by Mann-Whitney U test

To examine the types of leukocytes in these tissues, we stained the cells using different markers and analyzed by flow cytometer to identify the following cell types, namely helper T (TCRβ^+^ CD4^+^), and cytotoxic T (TCRβ^+^ CD8^+^), macrophages (F4/80^+^ CD11b^+^ Gr-1^+^), neutrophils (F4/80^−^ CD11b^+^ Gr-1^high^), and inflammatory monocytes (F4/80^−^ CD11b^+^ Gr-1^medium^) (Additional file 1). In the BALF from the *C. neoformans*–infected mice, active immune response was noted in all types of leukocytes examined, except CD8^+^ T cells demonstrated an average of 2-fold increment (Fig. 3a). No significant differences of the cell constituents were observed in lymph node (Fig. 3b). In spleen, the percentages of innate immune cells i.e. macrophages and neutrophils were increased at approximately 3– to 6–fold (Fig. 3c). On the contrary, percentages of T (both CD4^+^ and CD8^+^) cells were slightly reduced at 1.2– to 1.3–fold.

### Increased IL-17A–producing T cells in the lung of *C. neoformans H99*-infected mice

In the IL-17A–EGFP reporter mice, IL-17A–producing cells can easily be identified as they display EGFP fluorescence, hence this mouse model was utilized to determine the main source of IL-17A during *C. neoformans* infection. Our result showed that there was no profound increase of GFP^+^ cells amongst macrophage or inflammatory monocytes populations in the *C. neoformans*–infected mice (Additional file 2). The percentages of the IL-17A–producing neutrophils were scarcely increased in BALF (from 0.06 ± 0.03% to 1.08 ± 0.97%) and mLN (from 0.06 ± 0.03% to 0.62 ± 0.37), but not in the spleen.

**Fig. 4:**
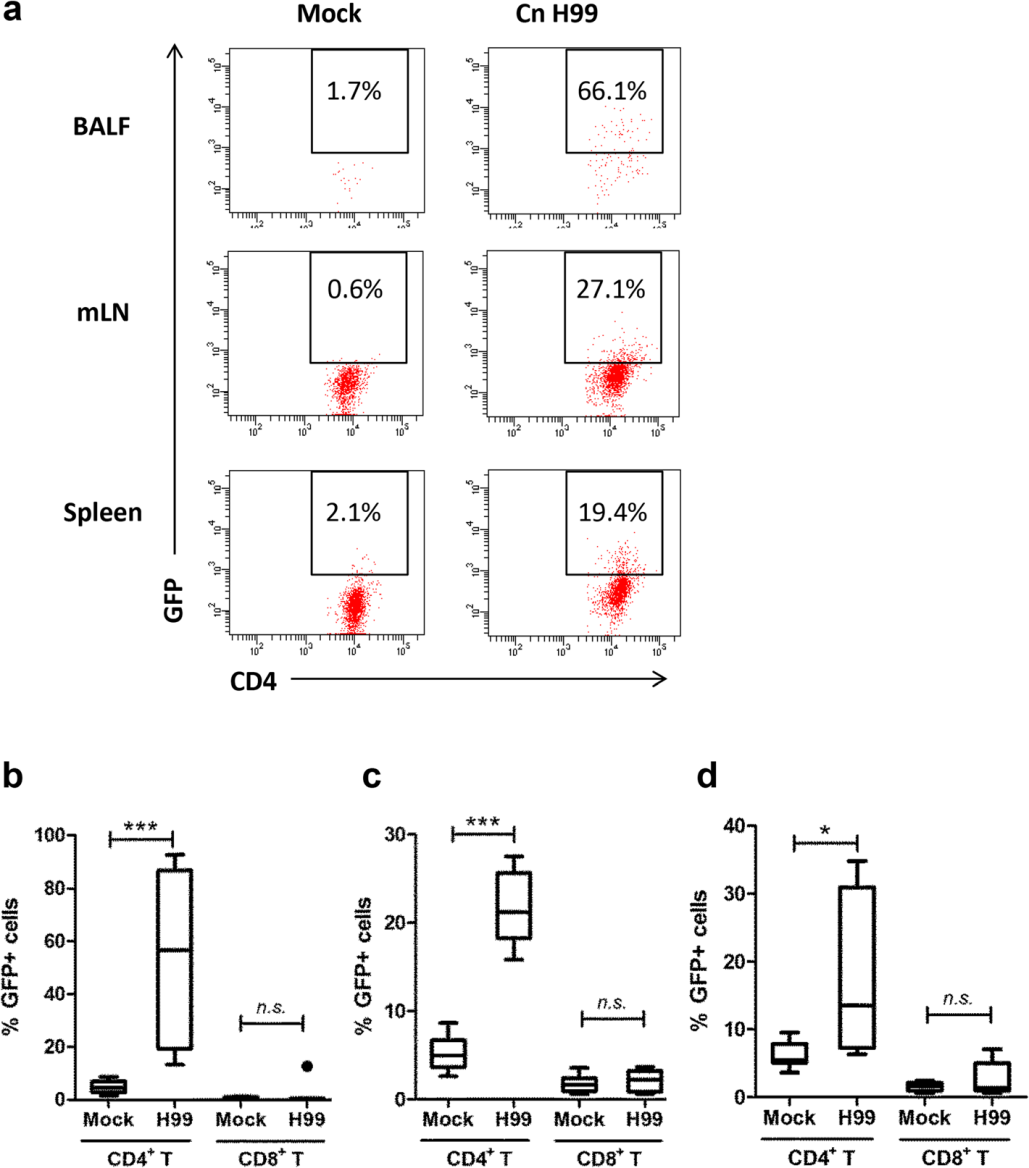
Production of IL-17A by CD4^+^ T helper cells. IL-17A–EGFP reporter mice (n = 4 per group) were uninfected (mock) or intranasally inoculated with 1 × 10^5^ cells with *C. neoformans* H99 strain (Cn H99), BALF, mLN and spleen were collected after 14 days for analysis. **a** A representative flow cytometrical plot of GFP^+^ cells in BALF, mLN and spleen among CD4^+^–gated T cells. % denotes the percentage of GFP^+^ cells appear inside the gated area. **b**–**d** Number of GFP^+^ cells among the CD4^+^– or CD8^+^–gated T cell populations in the (**b**) BALF, (**c**) mLN and (**d**) spleen. *P < 0.05, ***P < 0.001, *n.s.*: not significant or *P* ≥ 0.05, by Mann-Whitney U test

On the contrary, significant increases of GFP^+^ cells were detected among the T cells (Fig. 4). Major source of IL-17A was derived from TCRβ^+^ CD4^+^ but not TCRβ^+^ CD8^+^ T cells. Almost half (54.2 ± 11.6%) of the total lung infiltrated TCRβ^+^ CD4^+^ T cells recovered in BALF collected from *C. neoformans* H99-infected mice were GFP^+^, compared to only 4.8 ± 0.8% in the control (Fig. 4a, b). In the mLN, the percentages of GFP^+^ TCRβ^+^ CD4^+^ cells were approximately 4–fold greater at 21.7 ± 1.4% cells, compared to 5.1 ± 0.6% in control mice (Fig. 4a, c). The percentages of GFP^+^ cells among TCRβ^+^ CD4^+^ population were also marginally increased (17.53 ± 4.1%) among the splenic CD4^+^ T cells compared to 6.1 ± 0.6% in the control (Fig. 4a, d).

### IL-17A provides a supportive role in the immunity to pulmonary *C. neoformans* infection

**Fig. 5:**
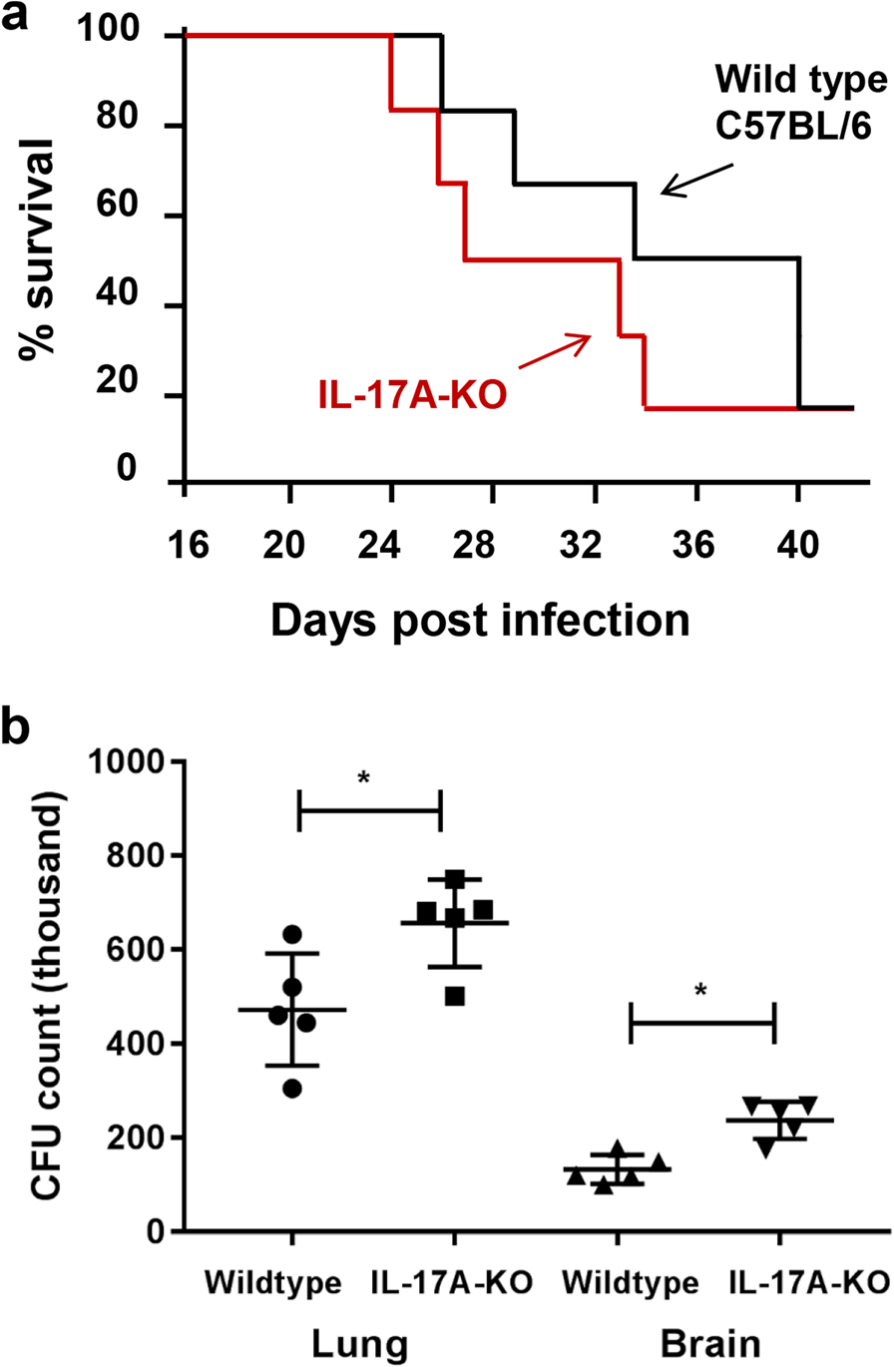
Attenuated protective immunity to *C. neoformans* in the IL-17A deficient mice. **a** Survival curve of the control and *C. neoformans*–infected mice. Wildtype C57BL/6 or IL-17A–KO mice (*n* = 6 per group) were intranasally inoculated with 1 × 10^5^ cells with *C. neoformans* H99 strain (Cn H99) and were observed closely over a peroid of 40 days. **b** Fungal burden of the *C. neoformans*–infected mice. Wildtype or IL-17A–KO mice (*n* = 5 per group) were intranasally inoculated with 1 × 10^5^ cells with *C. neoformans* H99 strain (Cn H99). Fungal CFU counts in the lung were quantitated after 14 days. Data is shown as mean ± SD. *P < 0.05 *n.s.*: not significant or *P* ≥ 0.05, by Mann-Whitney U test

A knock out mouse model was then applied to examine the protective role of IL-17A to host during *C. neoformans* infection. Intranasal inoculation of *C. neformans* in wildtype C57/BL6 mice and IL-17A–KO resulted in mice death starting from day 26 and 24, respectively. In IL-17A–KO mice, more than 80% (5 out of 6) mice died on day 34 whereas in wildtype control, this was observed at day 40 (Fig. 5a). The amounts of fungal cells in the local infection site (lung) as well as systemic infection (brain) were assessed. CFU counts in the lung derived from IL-17A–KO mice stayed at 657 ± 92, a higher level compared to 473 ± 119 in the wildtype control, whereas CFU count in the brain was 237 ± 39 compared to 133 ± 30 in the control mice (Fig. 5b). Hence, the absence of IL-17A may contribute to accelerated mice death as a result of an increased CFU count. Given that the differences in count were not substantial, further investigation is required to elucidate the factors contributing to the accelerated mouse mortality such as involvements of other types of immune cells and secretions of other cytokines or oxidative stress molecules.

## Discussions

Increasing prevalence of mortality attributed to cryptococcal meningitis in the immunocompromised patients underscores the importance of elucidating the host defense–pathogen interaction. In this study, we focused on (i) determining the expression and source of elevated IL-17A from different types of immune cells using a IL-17A–GFP reporter animal model and, (ii) investigating the role of IL-17A in protective immunity using a IL-17A–KO mouse model. Our data demonstrated elevated serum levels of T_H_17 cytokines, i.e. IL-17A, IL-17F and IL-23 in the infected wildtype C57BL/6 mice; and proposed the lung–infiltrating T_H_17 subset as the major source for IL-17A secretion at the lung upon pulmonary *C. neoformans* infection. In addition, we also showed that the absence of IL-17A resulted in a greater fungal burden and accelerated death of the infected mice, which may suggest a protective role of the potent IL-17A response at an early stage of *C. neoformans* infection.

Previous study suggests neutrophil as the predominant leukocyte in supplying IL-17A [17], however we showed here that the T_H_17 cells is the major player in secreting the cytokine, although the percentages of IL-17A–producing neutrophils were also increased. We consider EGFP reporter system as a more superior strategy to detect the intracellular cytokine production compared to fixation and staining method used in previous study. In fact, both neutrophils and T cells could have redundant or compensatory role as the depletion of neutrophils using anti-1A8 treatment in animal model causes an increased intracellular amount of IL-17A among the γδ^+^ T cell population [13].

Most studies thus far pinpoint the association of T_H_1-type cytokine responses with protective immunity against pulmonary cryptococcosis [18–20]. The cytokines in response were mainly those of T_H_1 subsets (IFN-γ, TNFα, IL-8), whereas moderate increases were also observed in T_H_2 (IL-4) and T_H_17 cytokines (IL-17A) [21]. A predominant T_H_1 and/or T_H_17 cytokine profile limits the growth of *C. neoformans* and *C. gattii*, whereas a T_H_2 cytokine profile promotes intracellular fungus proliferation [22]. In humans, it has also been reported that cryptococcal-specific CD4^+^ T-cell response is predominantly a T_H_1 type response with minimal involvement of T_H_2 and T_H_17 cells [21]. However, patients with higher IFN-γ or TNF-α production showed greater level of IL-17A level in their cerebrospinal fluid (CSF) [21]. These patients demonstrated lower fungal burdens and faster clearance of *C. neoformans* infection, suggesting that both T_H_1 and T_H_17 responses cooperatively provide optimal immunity against pulmonary cryptococcosis.

Previous study using IL-17A receptor (IL-17AR) deficient mice reported a slower rate of recovery from pulmonary fungal burden but the overall survival was not deteriorated [17]. By contrast, in this study, our data demonstrated accelerated death of the *C. neoformans*–infected mice. It is important to note that in IL-17AR deficient mouse model, the animal still possesses the ability to produce IL-17A, but signaling through its receptor, IL-17RA, is abrogated. Besides, it has also been shown that absence of IL-17A did not alter survival after 8 weeks of infection [15]. This could be due to differences of cryptococcal stains selected, whereby a highly virulent strain was used in our in vivo infection model compared to a moderate strain, 52D that was used in the previous study. Although previous study highlighted the robust activities of T_H_1 and T_H_17 assist in fungal clearance but lacks the efficacy in preventing dissemination of *C. neoformans* in animal infection [23], we report here that a higher fungal dissemination to the brain was observed in the surviving IL-17A–depleted mice. These data highlight that IL-17A participates in providing protective anti-cryptococcal host defenses through the suppression of fungal growth and dispersal.

This observation is also in line with studies on several other fungal species including *C. albicans* and *A. fumigatus* [7, 24, 25]. It was shown that a deficiency in IL-17A response results in increased susceptibility to oropharyngeal and disseminated candidiasis [7, 24]. Decreased neutrophil infiltration, increased fungal burden, and exacerbated pathology were reported upon IL-17A neutralization in *C. albicans* and *A. fumigatus* infections [7, 24, 25]. Toll IL-1R8 (TIR8), another negative regulator of T_H_17 response, has also been shown to reduce the susceptibility and immunopathology to candidiasis [26]. Some studies, on the contrary, provide evidence that outcome of aspergillosis in human is independent of T_H_17 responses [9], and the IL-23/IL-17A–driven inflammation could impede antifungal immune resistance and promote infection of *A. fumigatus* [27]. Hence, further investigation is necessary to validate the precise function of T_H_17 immunity towards fungal infection in humans.

## Conclusions

In summary, our data suggest that IL-17A derived from the lung infiltrating T_H_17 in BALF and mLN, plays a supportive role in rendering protection to pulmonary *C. neoformans* infection. Understanding the host immune response during cryptococcal infection is essential for the development of immunomodulatory therapies.

## Methods

### Fungal and cell culture

*C. neoformans var. grubii* (serotype A) H99 was obtained from American Type Culture Collection (ATCC). Environmental strains S48B, S68B and H4 were isolated from pigeon droppings, as described [28, 29]. All cells were stored at − 80 °C freezer until usage. To start the culture, a small drop of fungal cell stock was streaked on the Sabouraud’s dextrose agar (SDA) and incubated at 37 °C for 48 h. Then, 2 to 3 single colonies from freshly prepared agar plate were inoculated into Sabouraud’s dextrose broth (SDB) and incubated at 37 °C for 48 h.

### Animals

Wildtype C57BL/6, IL-17A–EGFP (*C57BL/6-Il-17a*^*tm1Bcgen*^*/J*) and IL-17A–KO (STOCK *Il-17a*^*tm1.1(ire)Stock*^*/J*) mice were obtained from Jackson Laboratory (Bar Harbor, ME). IL-17A–EGFP mice contain an IRES-EGFP-SV40-polyA signal sequence cassette inserted after stop codon of *Il-17a* gene and express EGFP as a marker of IL-17A activity. Whereas IL-17A–KO mice contained abolished IL-17A expression due to insertional mutation of a codon optimized Cre-recombinase and a polyA signal into exon 1 of *Il-17a* gene. Groups of 4 to 6 mice at age 8–12 weeks old were used in the study. All mice were maintained in individually ventilated cages under specific pathogen free condition. Mice were euthanized with CO_2_ inhalation when they exhibited overt signs including hunched posture, fur ruffling, weakness, increased respiratory rate and difficulty breathing. This study has been approved by the Faculty of Medicine Ethics Committee for Animal Experimentation at the University of Malaya (Reference number: 2013-12-03/MMB/R/EM).

### In vivo infection

Fresh cultures of *C. neoformans* were washed and harvested by centrifugation at 1800×g for 10 min. Cells were adjusted to 10^7^ cells/ml in phosphate buffer saline (PBS) using a hemocytometer. Mice were first anesthetized with intraperitoneal injection of a mixture of ketamine (90 mg/kg) and xylazine (10 mg/kg) before inoculated with intranasal pipetting of 20 μl (2 × 10^5^ cells) yeast suspension. For survival study, each infected mouse was examined daily from 2 to 6 weeks post infection. For other study, mice were euthanized with CO_2_ inhalation at 28 days post-infection and serum was collected from blood. Lung was lavaged with 1.0 ml PBS and BALF was collected. Lung, mLN, spleen and brain were excised for further analysis.

### Flow cytometry analysis

Cells collected from BALF, mLN or spleen were adjusted to 1 × 10^6^, washed with PBS-Tween20 with 3% fetal bovine serum, pelleted and stained with antibodies for 30 min. Two sets of antibodies used were (i) anti-TCRβ-PE (H57–597), anti-CD4-PE/Cy7 (GK1.5) and anti-CD8-PerCPCy5.5 (53–6.7), and (ii) anti-CD11b-APC (M1/70), anti-F4/80-PerCPCy5.5 (BM8) and anti-Ly-6G/Ly-6C-PE (Gr-1, RB6-8C5) (Biolegend, San Diego, CA). Cells were washed in PBS-Tween20 and resuspended in 1 ml flow buffer. Cells were analyzed in BD Canto II flow cytometer (BD Biosciences, Franklin Lakes, NJ) and data were processed using FACSDiva (BD Biosciences).

### CFU count

Brain and lungs from the mice were excised, weighed and homogenized in 1 ml PBS using glass slides. A total volume of 20 μl of the serially diluted homogenates (at 10, 100 and 1000 folds) were plated on SDA plates in duplicates and cultured at 30 °C for 48 h. Fungal load was quantified using colony forming unit (CFU) per ml by calculating yeast colonies on each plate.

### Bioplex assay

Sera from each mouse were collected for measurement of cytokines using Bio-plex Pro Mouse Th17 assay (Bio-rad, CA, USA) which included the following cytokines: IL-17A, IL-17F, IL-21, IL-22, IL-23, IL-31, IL-33 and MIP-3α according to the manufacturer’s instructions. The Multiplex bead working solution was diluted from 25× stock solution beads and 50 μl of it was added into each well followed by 50 μl of sample. Each cytokine standards and samples were assayed in duplicate as provided by manufacturer. Samples with microbeads were incubated at room temperature on a magnetic microplate shaker for 30 min. After incubation, Bio-Plex detection antibody working solution was then added, washed 3× with Bio-Plex wash buffer and finally 1× streptavidin-PE was added before reading the plate on the Bio-Plex 200 system (Bio-Rad). Cytokine concentrations from each tissue homogenates were calculated based on each cytokine standard curve.

### Statistics

All statistical analyses were performed using GraphPad Prism 6. Analyses between groups were performed using Student’s *t*-test or Mann-Whitney U test, whereby a *P* value of < 0.05 was considered statistically significant.

## Additional files

Additional file 1: Percentages of leukocytes in BALF, mLN and spleen of the *C. neoformans*–infected mice. Cells were stained with two sets of markers for identification of different cell types. (a) Gate P3: helper T (TCRβ^+^ CD4^+^), and gate P4: cytotoxic T (TCRβ^+^ CD8^+^). (b) Gate P3: macrophages (F4/80^+^ CD11b^+^ Gr-1^+^), gate P5: neutrophils (F4/80^−^ CD11b^+^ Gr-1^high^), and gate P6: inflammatory monocytes (F4/80^−^ CD11b^+^ Gr-1^medium^). (TIF 544 kb)

Additional file 2: Production of IL-17A by different innate cells after *C. neoformans* infection. IL-17A–EGFP reporter mice (*n* = 4 per group) were uninfected (mock) or intranasally inoculated with 1 × 10^5^ cells with *C. neoformans* H99 strain (Cn H99), BALF, mLN and spleen were collected after 14 days for analysis. (a–b) Number of GFP^+^ cells among the macrophages (F4/80^+^ CD11b^+^ Gr1^+^)–, neutrophils (F4/80^−^ CD11b^+^ Gr-1^high^)–, and inflammatory monocytes (F4/80^−^ CD11b^+^ Gr1^medium^)–gated cell populations in the (a) BALF and (b) mLN. *n.s.*: not significant or *P ≥ 0.05, by Mann-Whitney U-test. (TIF 260 kb)

## Abbreviations

BALF: Bronchoalveolar lavage fluid
EGFP: Enhanced green fluorescent protein
IL-17A: Interleukin-17A
KO: Knock out
mLN: Mediastinal lymph node
MOI: Multiplicity of infection
T_H_17: T helper 17
