# Supplementary figures and images for: Lung–infiltrating T helper 17 cells as the major source of interleukin-17A production during pulmonary Cryptococcus neoformans infection
Source: BMC Immunol. 2018 Nov 8;19:32. doi: 10.1186/s12865-018-0269-5 (PMC6225695; doi:10.1186/s12865-018-0269-5)

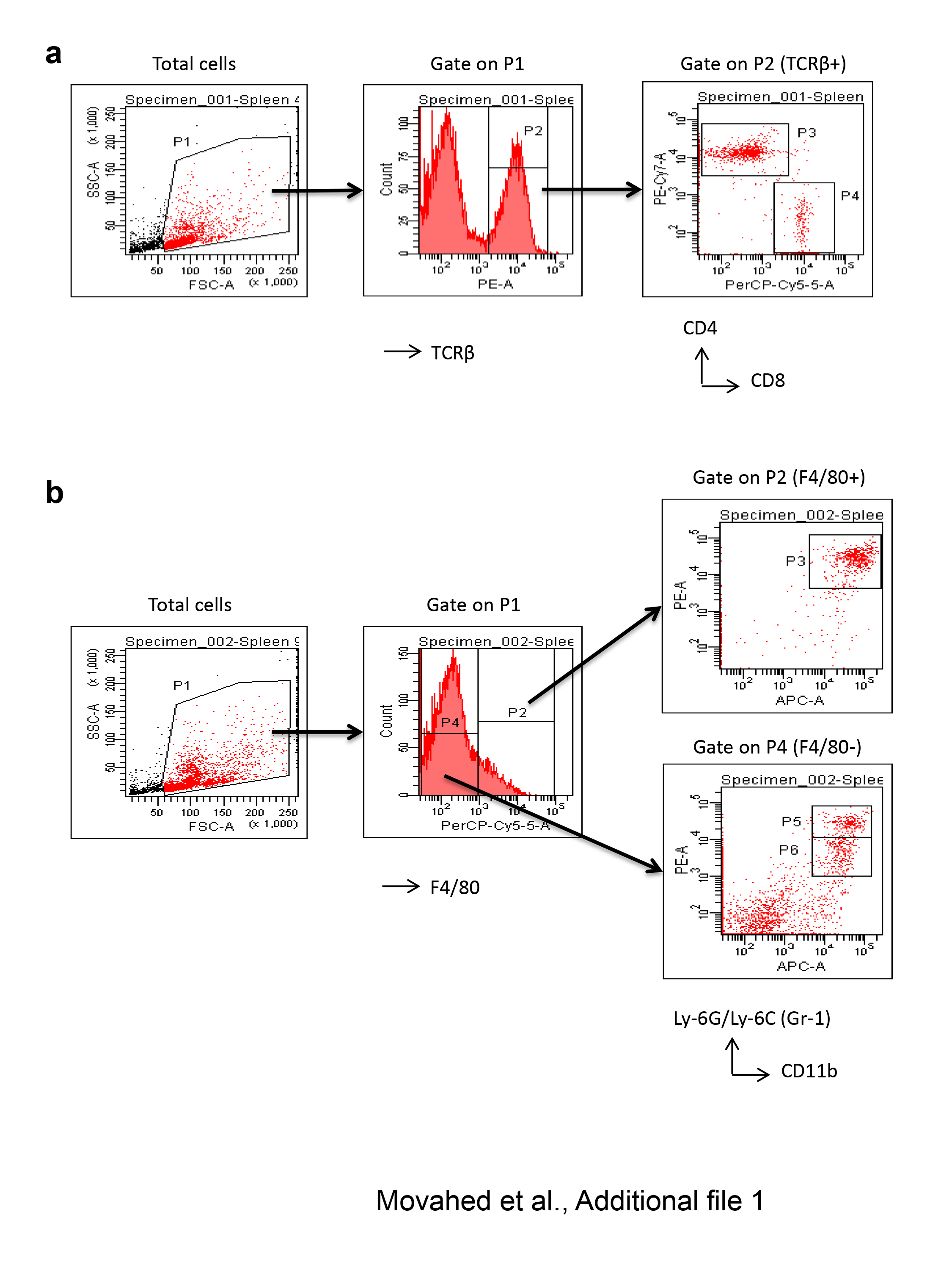

Supplement: Supplementary file 1 — Percentages of leukocytes in BALF, mLN and spleen of the C. neoformans–infected mice. Cells were stained with two sets of markers for identification of different cell types. (a) Gate P3: helper T (TCRβ+ CD4+), and gate P4: cytotoxic T (TCRβ+ CD8+). (b) Gate P3: macrophages (F4/80+ CD11b+ Gr-1+), gate P5: neutrophils (F4/80− CD11b+ Gr-1high), and gate P6: inflammatory monocytes (F4/80− CD11b+ Gr-1medium). (TIF 544 kb) [file 12865_2018_269_MOESM1_ESM.tif]

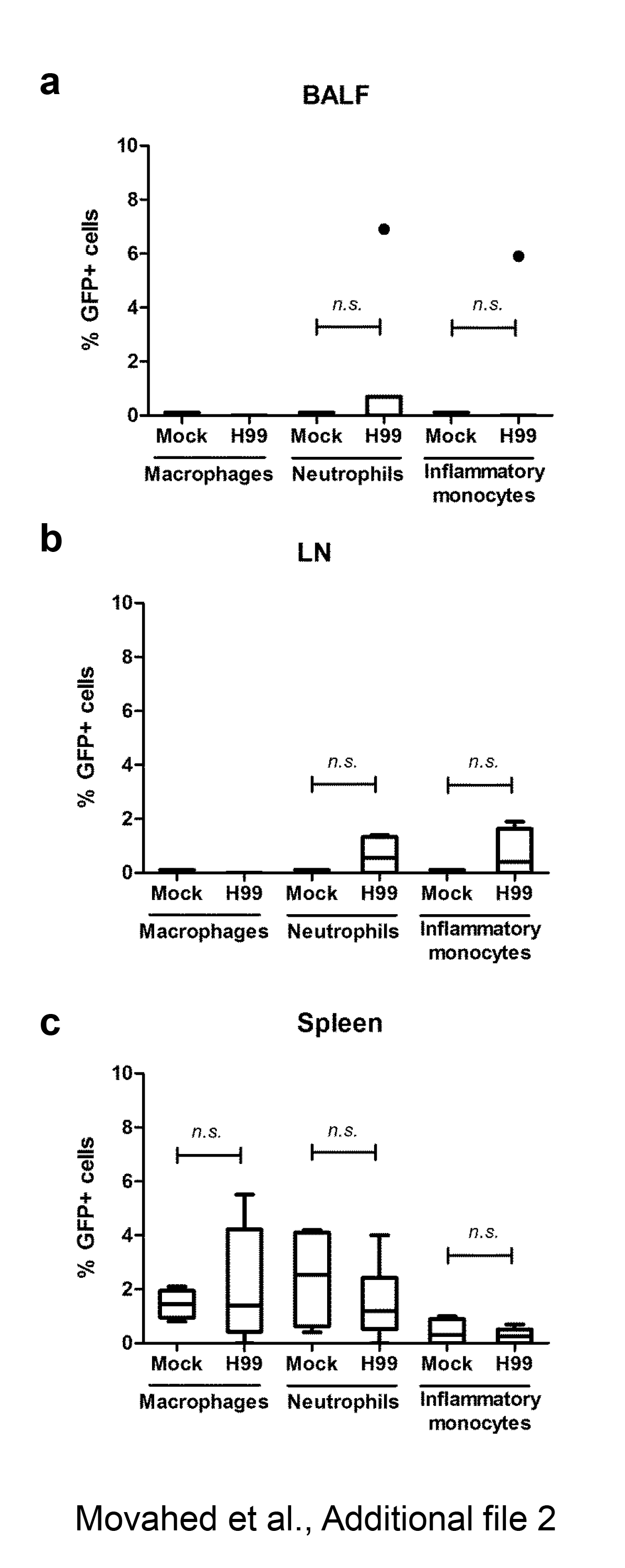

Supplement: Supplementary file 2 — Production of IL-17A by different innate cells after C. neoformans infection. IL-17A–EGFP reporter mice (n = 4 per group) were uninfected (mock) or intranasally inoculated with 1 × 10^5^ cells with C. neoformans H99 strain (Cn H99), BALF, mLN and spleen were collected after 14 days for analysis. (a–b) Number of GFP+ cells among the macrophages (F4/80+ CD11b+ Gr1+)–, neutrophils (F4/80− CD11b+ Gr-1high)–, and inflammatory monocytes (F4/80− CD11b+ Gr1medium)–gated cell populations in the (a) BALF and (b) mLN. n.s.: not significant or *P ≥ 0.05, by Mann-Whitney U-test. (TIF 260 kb) [file 12865_2018_269_MOESM2_ESM.tif]
